# ICCD camera technology with constant illumination source and possibilities for application in multiwavelength analytical ultracentrifugation†

**DOI:** 10.1039/c8ra08752k

**Published:** 2018-12-05

**Authors:** Joseph Pearson, Helmut Cölfen

**Affiliations:** University of Konstanz, Physical Chemistry, Department of Chemistry, Universitätsstraße 10 Konstanz 78457 Germany helmut.coelfen@uni-konstanz.de

## Abstract

A new concept for multiwavelength detection in analytical ultracentrifugation is demonstrated, based on the technology of an Andor iStar intensified CCD camera and constant illumination source. The camera is coupled to an Andor Shamrock spectrograph providing a spectrometer system capable of UV/vis acquisitions with wavelengths from 190 to 790 nm. The details of the camera functions are described, and the essential operational modes demonstrated with proof of principle measurements. The Andor spectrometer system is proven to be much more sensitive than previous AUC detectors. In fact, it is so sensitive in high gain modes that signal quality is limited by photon shot-noise. Acquisition strategies are presented for detection systems working with low-light illumination sources, and the necessity of overcoming shot-noise limited signal quality is revealed. Several illumination optical concepts are tested and it is shown that when sufficient illumination is applied the detection system is capable of outperforming commercial and academic detectors previously reported in terms of signal-to-noise. A path forward for overcoming the remaining challenges is provided. The findings in this manuscript will be applicable to any type of high sensitivity detector design.

## Abstract 2

A new concept for multiwavelength detection in analytical ultracentrifugation is demonstrated, based on the technology of an Andor iStar intensified CCD camera and constant illumination source.

## Graphical abstract

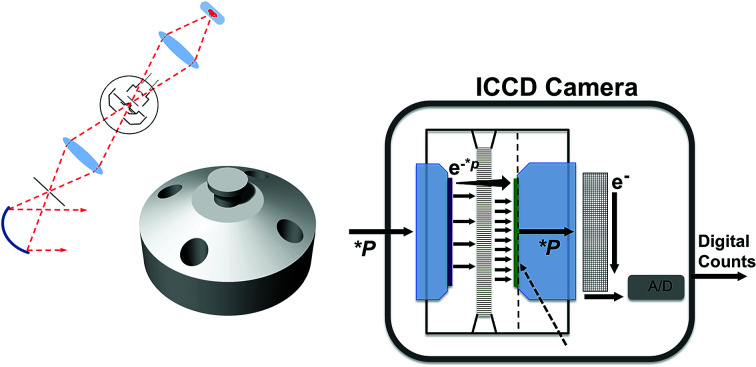

## 1. Introduction

Recent years have brought new developments in ultracentrifuge technology, such as the introduction of the Centrifugal Fluid Analyzer (CFA) from Spin Analytical, opening up new opportunities for analytical ultracentrifugation (AUC) detector development. The CFA platform is based on the concept of a smaller vacuum chamber sized to accommodate only the rotor, with windows for optical signals on three separate tracks. Detectors may then be mounted above and below the vacuum chamber, and are not limited by space or vacuum compatibility. This design was already realized in the first commercial analytical ultracentrifuges from decades ago, such as the Beckman Model E, MSE Centriscan or the MOM ultracentrifuge.^1^ Placing the detectors outside of the vacuum chamber allows for the possibility of an entirely new class of detectors, and is the inspiration for the multiwavelength (MWL) absorbance detector outlined here. The Open AUC MWL absorbance detectors previously described have been designed to work within the Beckman-Coulter preparative style centrifuges.^2–6^ The detectors are mounted in the centrifuge vacuum chamber and light is introduced *via* optical fibers. This imposes hardware constraints, and performance limitations as previously documented.^4^ All open source and commercial absorbance detectors of the last several decades have been based on the utilization of a xenon flash lamp for rotor period synchronization. Charge Coupled Device (CCD) sensor technology, as implemented in the Open AUC MWL spectrometer, does not have a charge transfer rate fast enough to allow for rotor synchronization at high speed. Furthermore, the luminance provided by a pulsed xenon lamp is typically much higher than a constant illumination arc lamp. While the xenon lamp is able to provide rotor synchronization with high intensity light pulses, it is also the limiting factor in reducing stochastic signal noise due to flash-to-flash variability.^4^

In the following pages we analyze the feasibility of a MWL detector based on an iStar Intensified Charge Coupled Device (ICCD) camera and 193i Shamrock spectrograph from Andor Technology Ltd. The ICCD camera technology allows for both high sensitivity detection and optical gating, in addition to the triggering electronics necessary for rotor period synchronization. The unique characteristics of ICCD sensors therefore provide the opportunity to implement a high stability constant illumination source. A few options exist for constant illumination sources emitting from the UV through the visible. A xenon arc lamp provides high visible light emission, but negligible output in the UV. A xenon–mercury arc lamp provides UV emission, however, the output is highly non-linear showing enormous emission spikes at selected wavelengths in the UV and visible, and is therefore unsuitable for the spectrometer based detection system. The system tested here makes use of a combination deuterium–tungsten (D–W) mirror based illumination assembly. The illumination of a D–W assembly is significantly lower than xenon or xenon–mercury arc lamps, but is able to provide balanced spectral output from the UV through the visible.

## 2. Methods

### 2.1 Andor spectrometer

A spectrometer consists of two principle elements; a dispersive element for splitting light into spatially separated wavelengths, known as a spectrograph, and a sensor for spatially dependent detection of the respective wavelengths. The spectrometer system used here is composed of an iStar ICCD 2D array camera (DH320T-25F-04) with Gen 2 Intensifier and Shamrock 193i spectrograph from Andor Technology Ltd., where the camera's horizontal pixel array captures the spectral domain, and the vertical pixel array images the spatial dimension along the length of the spectrograph entrance slit. The specifications of an Andor spectrometer system are significantly different than the Open AUC MWL detectors previously reported on. An outline is provided in the ESI Table 1,† showing a comparison to the basic features of an Andor spectrometer and D–W lamp, with the Ocean Optics USB2000+ and xenon flash lamp used in the Open AUC MWL system.^4,7^

The most noteworthy attributes of the Andor spectrometer system are the high sensitivity, and the optical gating available with the ICCD camera, allowing for rotor synchronization and therefore implementation of a constant illumination source. In addition, the camera is sensitive deeper into the UV, and the spectrograph allows for interchangeable gratings for adapting to a wide spectral range.

### 2.2 2D-imaging

**Fig. 1:**
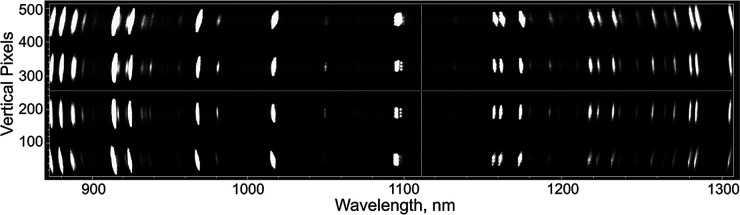
Image of four vertically separated fiber bundles, each containing three 100 μm fiber strands, captured with an iStar camera and Shamrock 193i spectrograph. Image used with permission from LOT-Quantum Design GmbH.

The original design proposal involved using the 2D imaging array of the Andor spectrometer system to capture the spectral profile of an entire MWL AUC cell channel in a single acquisition, where the spectral dimension is recorded across the horizontal pixels, and the radial channel dimension is recorded in the vertical pixel dimension. However, test data provided by the Andor distributor, LOT-Quantum Design GmbH, showed that the vertical resolution of the spectrometer system was not adequate to meet the radial resolution requirements for AUC data; typically <50 μm per radial increment. The test image in Fig. 1, shows 100 μm fiber strands may be discriminated in a three-fiber bundle only at the center wavelength of the spectrometer imaging array.

This experimental evidence demonstrated that high resolution imaging incorporating a diffraction grating is not yet feasible with present technology. Subsequent investigations explored the performance of the spectrometer when arranged in a scanning mode. For this design, the spectrometer is mounted in a scanning track assembly, similar to the design typically employed in Open AUC MWL detectors.^4^ The acquisitions only incorporate a portion of the vertical CCD array corresponding to the spatial width of a sample channel, however the unused portion of the array may still be utilized in a high-speed acquisition scheme, and is described below.

### 2.3 ICCD camera

**Fig. 2:**
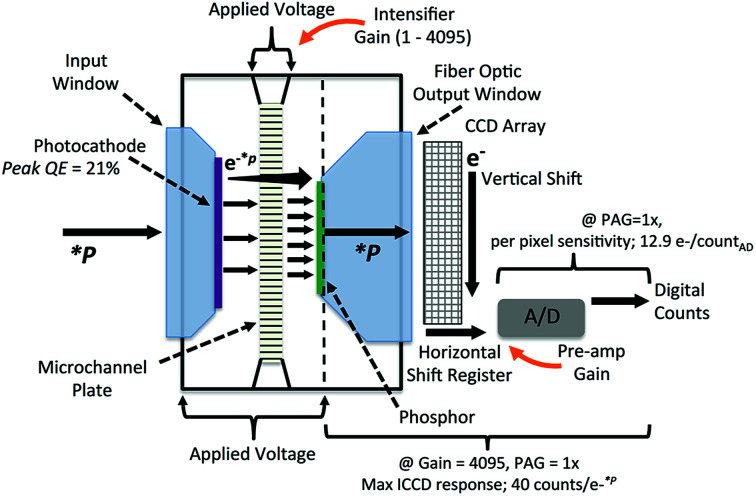
Illustration of construction and operational principles of an Andor CCD intensifier. Some specifications provided are unique for the device configuration utilized in this study.

The intensifier of an ICCD camera is installed in front of the CCD detector, and is composed of several elements including a photocathode, micro-channel plate (MCP), phosphor screen and fiber optic output window.^7^ The basic construction of the intensifier is illustrated in Fig. 2.

As depicted in Fig. 2, illumination photons, *P, enter through the input window and strike the photocathode. The photocathode then emits photoelectrons, e^−^*^p^, towards the MCP, drawn by an electric field. The MCP is composed of a honeycomb of 6–10 μm diameter glass channels, with a high potential applied across the length of the channels. The potential is adjustable, and referred to as the intensifier gain. As photoelectrons are accelerated down a channel, more photoelectrons are dislodged in an amplification cascade. The cloud of electrons, e^−^, emerging from the MCP then strike the phosphor coating on the inside of the fiber optic output window, and are converted back to photons. It is important to note that for fast frame rate applications such as described here, a P46 phosphor with 100 ns decay time is required. The fiber optic output window guides the photons onto the 2D CCD camera pixel array, where the photons are again converted into electrons. This application uses a fiber optic taper selection providing 1× magnification. During readout the electrons in the CCD pixels are shuttled vertically into the horizontal shift register, and then read out sequentially to the analog to digital converter (A/D). The A/D incorporates 16 bit architecture, providing counts from 0 to 65 536, and includes a pre-amplifier gain (PAG) option selectable in software. The electrons entering the A/D make-up the voltage signal that is converted from analog form into digital counts recorded in the software.

The intensifier gain is a 12 bit, 0 to 4096, increment setting in the software that adjusts the voltage to provide signal amplification through the MCP. However, the camera circuitry may also reverse the polarity of the gain voltage, whereby preventing photoelectrons from traveling into the MCP. With this mode, the intensifier rejects nearly all photon signals entering the camera, effectively acting as an electro-optical gate with sub-nanosecond time resolution. This functionality is the basis for ICCD synchronization in AUC. The digital circuitry for electronic gate operation is contained within the camera housing, and is fully integrated in the software driver. Furthermore, digital delay generator circuitry is also included, providing trigger signals for up to three external devices.^7^

The intensifier gating is completely independent from the CCD array readout. This allows for use of a so-called ‘integrate on chip’ mode, where the intensifier gate is opened and closed multiple times, during a single CCD acquisition. This operating mode is analogous to the ‘flash accumulation’ method previously employed with pulsed light sources in MWL AUC optics.^4^ However, contrary to the xenon flash lamp based detectors, using a constant light source allows signal integration by modulation of the duration the intensifier gate is open, known as the gate width. The width may then be set to match the time required for the sample channel to rotate past the detector position. This is essential for collecting sufficient signal. Furthermore, the nanosecond response of the intensifier gating means the repetition rate is limited only by the rotational speed of the rotor.

To a first approximation the signal-to-noise ratio (SNR) of a CCD camera may be estimated by the following eqn (1);1
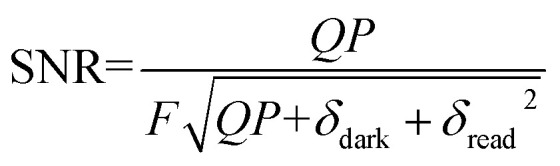
where *Q* is the quantum efficiency of the intensifier, *P* is the number of photons, *δ*_dark_ is the dark current of the CCD, *δ*_read_ is the readout noise, and *F* is a multiplier characteristic to ICCD detectors and equal to 1.6.^7^ Ignoring dark current, readout noise, and assuming a completely full horizontal shift register pixel, *QP* = 584 300 electrons, a theoretical maximum SNR = 478 may be calculated. However, this does not indicate that a SNR near this value will be achieved in practice, as the theoretical maximum assumes 100% quantum efficiency through the detector. Actual sensitivities as a function of gain settings are required to predict the shot-noise characteristics. The sensitivities of the intensifier are individually measured and reported for each camera system as a function of gain settings, and are not available in the manufactures catalog specifications. Predicting the SNR for a camera system is therefore difficult without knowledge of the illumination power, *i.e.* the number of photons that may be collected during an acquisition. A detailed examination of the noise characteristics for the iStar-Shamrock system tested here is provided in the results to follow.

It is important to note that when operating the camera with a PAG setting of 1×, the internal sensitivity converts to 12.9 e^−^ per count, allowing the full capacity of the readout register to be utilized, whereas when operating at a PAG setting of 4× the A/D saturates first at 65 535 counts, before the maximum pixel well capacity is reached. This means that the full pixel well capacity, 584 300, is reached at 45 300 A/D counts when operating with 1× PAG setting.

### 2.4 Illumination optics

**Fig. 3:**
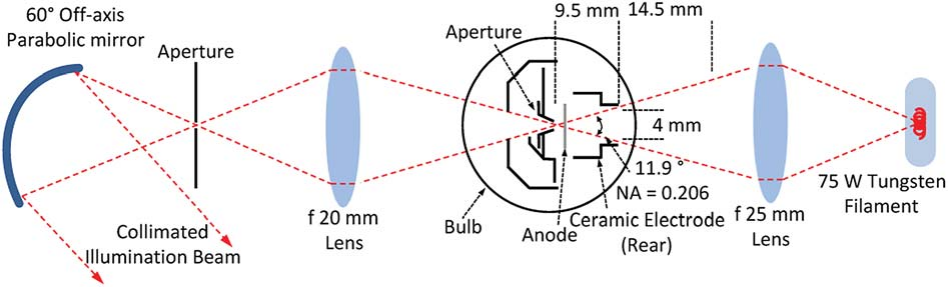
Illustration of illumination optics assembly utilizing a Hamamatsu X2D2 ‘see-through’ deuterium lamp and tungsten filament lamp. Note; not drawn to scale.

As discussed in the introduction, limited possibilities exist for UV/vis illumination sources. The work here explores the feasibility of a 26 W X2D2 high brightness deuterium lamp from Hamamatsu, with ‘see-through’ type construction, in combination with a 75 W tungsten filament lamp. A standard Bosch automotive 12 V H7 tungsten lamp is used with a Statron type 2231 galvanically isolated power supply. A Hamamatsu type M9521 regulated voltage supply powers the Hamamatsu X2D2 lamp. The lamps were shown to have comparable noise levels, data not shown. It is important to note that the power rating of the lamp is related to the electrical load and is not a direct measure of the illumination intensity emitted. The ‘see-through’ architecture of the X2D2 deuterium lamp allows for construction of an optical system, where illumination from the tungsten lamp is focused through an aperture located at the point of arc generation of the deuterium lamp, as depicted in Fig. 3. The X2D2 arc point thereby acts as a simultaneous point source of UV illumination from the X2D2, in addition to visible light originating from the tungsten source.

In order to allow for additional space between the X2D2 lamp and the collimating element, an additional lens is positioned after the lamp, and the light is refocused through a 0.5 mm pinhole aperture. An off-axis parabolic mirror is then used to collimate the UV/vis illumination beam.

The numerical aperture and diameter of the X2D2 lamp limits the amount of light that may be focused into the lamp from the tungsten source, and furthermore, how much UV/vis light emerging from the X2D2 lamp that can be collected. Stock 25 mm diameter Qioptiq quartz lenses were selected for the focusing system within the illumination assembly. Winlens 3D basic from Qioptiq is a convenient tool for modeling simple optical designs, and is used to select suitable lens elements. A 25 mm focal length lens allows for a sensible focusing scheme, where the approximately 1 mm wide tungsten filament is imaged onto the center of the X2D2 arc point, with a magnification of −0.5, while including the entire cone of light possible with the X2D2 lamp aperture. The fractional magnification means that the 1 mm wide tungsten filament is imaged to match the 0.5 mm arc point aperture of the X2D2 lamp.

The light emerging from the X2D2 lamp is again refocused through a 0.5 mm pinhole aperture. This allows for the subsequent light collimation element to be placed closer to the point source without being blocked by the edge of the lamp. It also allows for adjustments to be made to the numerical aperture of the incident light cone, such that an optimal collimation beam may be generated. A 20 mm quartz lens is found to provide adequate focusing. The lens models generated in Winlens 3D is illustrated in ESI Fig. 1.† Exact positioning of the lenses did not show a significant effect on the light intensity output of the optical system. Therefore, while a more complicated illumination system, with compound lenses for example, may provide higher power throughput optimization, it would likely be only a nominal improvement.

The light cone emerging from the pinhole aperture is collimated by a 60° off-axis parabolic mirror from Edmund Optics, with 33.85 mm effective focal length. Chromatic aberration from the illumination system before the pinhole aperture will manifest as an illumination intensity variation across the collimated beam front. This will arise from both the lenses used in addition to temperature variations across the arc and filament. However, the mirror element assures that all rays reflected off the parabolic surface will collimate regardless of color. By selection of a 60° off-axis parabolic mirror, a compromise is found for generating a high quality collimated beam with a diameter matching the dimension necessary to illuminate an entire AUC cell channel; approximately 14 mm. In general, lower angled off-axis parabolic mirrors will collimate with less aberration than angles approaching 90°. However, at very low angles a long focal length is required so the beam is not blocked by the mechanics of the illumination assembly. This would then produce a beam with a diameter wider than the required sample field, and therefore a loss of illumination intensity.

The signal response of the camera will be determined by multiple factors including the photon flux produced by the lamps, efficiency of the optical assembly and the sensitivity of the camera. The illumination assembly aims to produce a beam of light with maximum photon flux, while minimizing collimation divergence. Low beam divergence is required for good radial resolution across the AUC sample channel. In general, an inverse correlation is always observed between beam intensity and collimation quality, where optimization requires a compromise between the two.^4^ The size of the pinhole aperture is one convenient parameter for sensitive modulation.

The results detailed below examine the SNR characteristics of the spectrometer system and the dependence of the illumination assembly described above. It will be shown that the previously described illumination assembly is inadequate for providing a high SNR. To demonstrate the necessary improvements in light throughput, two additional arrangements are tested. First, a 60 mm focal length cylinder lens is introduced after the parabolic collimating mirror to produce a line of illumination focused on the plane of the spectrometer entrance slit. The line was arranged orthogonal to the slit length, simulating illumination of the sample channel. Line-focused illumination is similar to what is achieved in the Beckman-Coulter XL-A AUC, albeit by a much different optical scheme.^8^ Second, the cylinder lens is replaced by a 60 mm focal length bi-convex lens, producing a spot of illumination focused on the plane of the spectrometer entrance slit. Spot-focused illumination is equivalent to the confocal type optics described previously for Open AUC MWL detectors.^4,9^ A schematic of the test setup is illustrated in the ESI Fig. 2.† These two additional lens possibilities are of course not ideal because of the chromatic aberration inherent to refractive elements, but are presented to demonstrate proof of principle in bench tests. An in-centrifuge test would require the use of off-axis parabolic or cylindrical mirrors to accomplish the optical transformation that is being demonstrated with lenses here. The advantages and drawbacks of various illumination schemes have been discussed previously.^4^

Compact imaging optics were designed based off the concept of mirror imaging optics published previously.^4,9^ The design details and basic imaging tests are provided in ESI Fig. 3 and 4.† However, the following results are primarily concerned with the illumination source, and detector performance, and are therefore conducted without imaging optics, where the beam is directly focused onto the entrance slit of the spectrometers. In this way the results are not convoluted with the efficiency of additional elements in the beam path. In practice, losses from the measurement cell windows and imaging elements are typically on the order of 10 to 15%.

## 3. Results

### 3.1 Spectral sensitivity

The spectrum acquired from the iStar – 193i Andor spectrometer system is highly adaptable and tunable. The 193i Shamrock spectrograph features an exchangeable grating turret where two separate gratings may be mounted simultaneously. The turret then rotates by software command to change between gratings. The center wavelength position of the gratings may also be adjusted to tune the grating efficiency towards higher or lower wavelengths. A six-position filter wheel is also included, and operates by software command. Selection of appropriate filters is necessary to remove higher-order diffraction wavelengths when measuring at higher wavelengths. The entrance slit of the spectrograph is manually adjustable. The width of the slit affects the spectral resolution from the grating, in addition to the transmission efficiency of the spectrograph. However, a width opening should be set to match the spatial resolution of the optical system and the radial resolution requirements of the experimental data. The slit width is fixed at 25 μm for the tests conducted here.

**Fig. 4:**
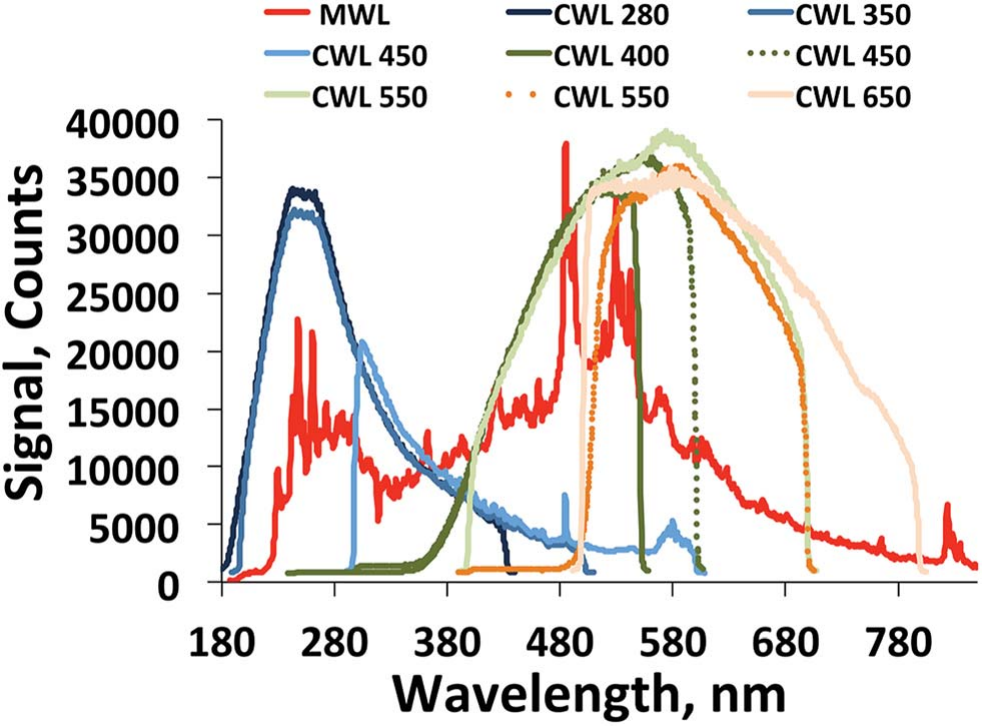
Spectral sensitivity of an iStar – 193i Andor spectrometer system configured with 400 lines per mm gratings, with 250 nm and 550 nm blaze wavelengths. Effect of center wavelength adjustment is shown in the plots. A typical spectral acquisition from a USB2000+ and xenon flash lamp used in the Open AUC MWL detector is shown in red for comparison.

Two 400 lines per mm gratings, with 250 nm and 550 nm blaze wavelengths, were selected for testing, with the aim of providing signal from the deep UV to the NIR. The spectrometer signal recorded is dependent on the illumination intensity spectrum, wavelength efficiency of the grating, and spectral sensitivity of the detector. The spectral response for the X2D2 lamp with UV optimized grating, and tungsten lamp with visible optimized grating is shown in Fig. 4.

The X2D2 lamp and UV grating is shown to provide excellent signal deep in the UV, with 8000 counts at 200 nm. The useable spectral range extends up to approximately 400 nm, and adjustment of the center wavelength positioning does not show a significant effect on the useable spectral range. The tungsten lamp and visible grating provides useable signal extending from approximately 400 nm to 790 nm. Adjustment of the center wavelength allows the spectrum to extend slightly further into the red. The implementation of a 500 nm high pass filter is also shown, however, this filter choice is clearly not a sensible option for this arrangement, as the useable spectrum drops off at 790 nm. A 400 nm filter would be a more logical choice. The results show that while the gratings selection has regions of good signal response, they are a suboptimal configuration for all UV/visible acquisitions. However the grating turret is easily interchangeable, so that alternative grating combinations may be installed to optimize for the spectral region of interest. The Gen 2 intensifier used here is specified to provide signal from 180 to 850 nm, and will limit extending the spectral range. Intensifiers with efficiency profiles extending to longer wavelengths are also possible, but will compromise the UV signal. Broad-spectrum acquisitions, equivalent to the Open AUC MWL, cannot be recorded at one time due to the size of the CCD chip, the type of gratings used and geometry of the spectrograph. However the two installed gratings may be exchanged programmatically, and overlapping spectra may be ‘stitched’ together in software to achieve broader spectra.

### 3.2 SNR

The effective pixel size of the CCD chip is 25 μm and the DH320 camera head has 1024 × 255 pixels. The spectrometer entrance slit is oriented such that 255 pixels span the length of the slit, covering 6.375 mm. A standard AUC sample channel is 12 to 14 mm long with a 2.5° sector shape. Optical scanning devices in AUC typically have a radial resolution of approximately 25 μm, and length in the tangential dimension of 1 mm.^9^ Therefore, only a 1 mm segment of the iStar CCD chip, equating to 40 pixels, is necessary for an acquisition. The camera driver allows for binning pixels in both the vertical and horizontal dimension as they are shifted into and out of the horizontal shift register. Binning integrates the electrons in the pixel wells before readout through the A/D. With 400 l mm^−1^ gratings being used, up to 4 pixels may be binned in the horizontal dimension while remaining within the spectral resolution specification of 1.4 nm.

The sensitivity of the camera may be adjusted by two software selectable parameters; the pre-amplifier gain (PAG) and the intensifier gain. The amount of signal collected may then be modulated by the duration the intensifier is activated for, known as the gate width. A first test of the iStar camera sensitivity was made by comparison with the Ocean Optics USB2000+ spectrometer, using an Ocean Optics 7.5 W USB-DT deuterium–tungsten lamp. The USB-DT is a typical lamp used for spectrophotometric measurements with the USB2000+, and allows for a relative comparison of spectrometer sensitivities, because a calibrated light source was not available. Moreover, the 26 W X2D2 lamp is too powerful to use with the USB2000+ detector because the pixels will be saturated at even the lowest possible integration setting of 1 ms. A 500 μm fiber optic cable connected the lamp and USB2000+ spectrometer. The integration time of the spectrometer was then adjusted such that a signal over 50 000 counts was detected at 530 nm. A signal of 50 000 counts means that over 80% of the pixel capacity is being used. To achieve this, the integration time required is 155 ms. The same illumination assembly was then applied to the Andor spectrometer system. The exit tip of the fiber optic cable was placed at the entrance slit of the spectrometer. The camera vertical binning was then set to 60 to approximately match the pixels illuminated by the fiber optic cable, and 4 pixels were binned horizontally. With the PAG set to the 4× maximum, and the intensifier gain set at 4000, only 600 μs are required to achieve 50 000 counts, demonstrating the amazing sensitivity of the intensified CCD camera.

**Fig. 5:**
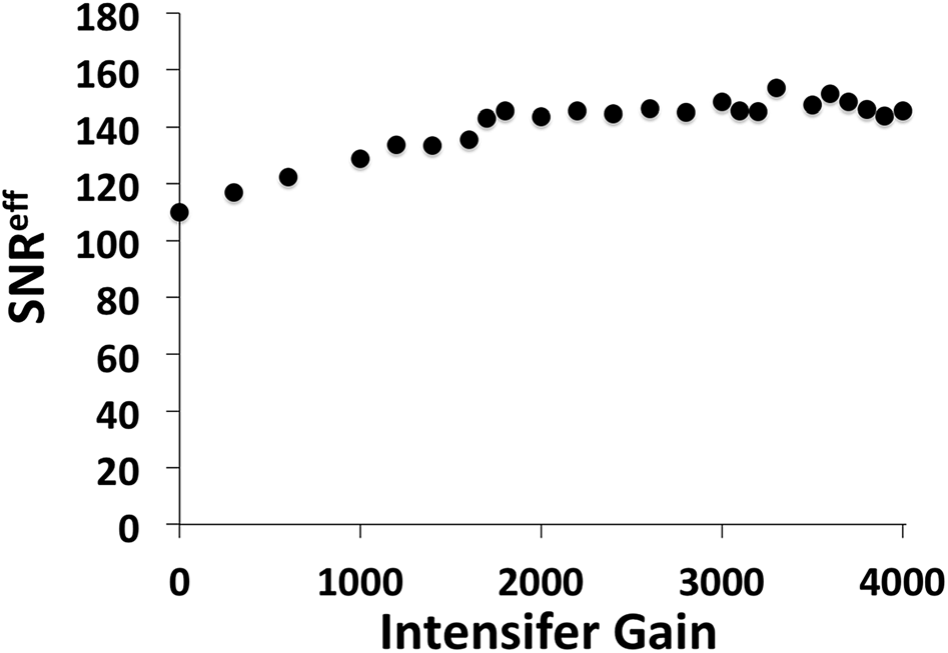
Plot of SNR^eff^*versus* intensifier gain, from an Andor iStar ICCD camera, where SNR^eff^ is computed by scaling *N*^1/2^ averages over 155 ms acquisition time.

The experimental SNRs of the cameras were then compared by computation of the ratio of average signal to standard deviation for 500 consecutive acquisitions. With the same illumination and camera configurations, the USB2000+ provides a SNR of 210, whereas the Andor system shows a SNR of only 14.8. Because the Andor camera was able to make the identical acquisition in a small fraction of the integration time required by the USB2000+, direct SNRs are not a meaningful comparison. Alternatively, the Andor camera could be presumed to collect, *N*, repetitive measurements over the 155 ms duration of a USB2000+ acquisition, and SNR improvement due to averaging is considered. The SNR is known to increase with *N*^1/2^, and the number of possible acquisitions may be calculated by division of the gating width time. This allows for a simple conversion to effective SNR, SNR^eff^, for a given gain and width setting, and corresponding SNR. At lower gain, a longer width is required, and therefore provides a higher SNR, but fewer repetitions may be averaged. A plot of SNR^eff^ for various intensifier gain settings is shown in Fig. 5.

At all gain settings the Andor spectrometer system is shown to provide a lower SNR than the Ocean Optics USB2000+. To understand why this is the case, it is necessary to consider the shot noise contribution as expressed in eqn (1). The sensitivity of the camera as a function of gain and PAG settings is given in the documentation shipped with the spectrometer system. At a high gain of 4000 and 4× PAG setting, the counts per photoelectron, e^−^*^p^, received through the intensifier is approximately 120, from measurements provided in the calibration documentation that is shipped with the spectrometer. Therefore the counts detected by the camera may be converted to e^−^*^p^, and thereby provide the basis for a calculation of shot-noise. For example, if 50 000 counts are detected, this equates to 416 e^−^*^p^ and, following eqn (1), a SNR of 12.7 is calculated. This is close to the SNR of 14.8 as reported above. Readout noise and dark noise may be estimated by the specifications of the camera, but only produce approximately 1% lower SNR, and therefore are acceptable to neglect in this comparison. The calculations here verify that the camera, operating in high gain mode, while sensitive enough to acquire adequate signal from a constant source lamp over the short time that a spinning sample is available, is in fact so sensitive that it is limited by shot-noise. The shot-noise regime is a fundamental limit imposed by the stochastic nature of discretized photon illumination. The shot-noise fluctuations follow Poisson statistics and cannot be overcome.^10^

There are two possibilities to compensate for shot-noise; collecting more photons and averaging sequential acquisitions. Collecting more photons requires reducing the gain of the intensifier and extending the signal integration time, but in order to synchronize with the spinning rotor, the gating width must be set to match the channel timing. However, the accumulation cycle period may be extended and the intensifier triggered sequentially to capture multiple rotor revolutions in a single acquisition. The extent to which integral signals may be accumulated is limited by the scan time tolerance of an AUC experiment. The same will be true for the level of averaging that may be applied.

Scan time tolerance is partly dependent on the nature of the AUC experiment being conducted, and the detail of information being extracted from the data. For a typical AUC experiment, where sample sedimentation occurs over several hours, scan times are conservatively kept below 60 seconds.^3^ Slower sedimentation processes may extend the scan period without a significant distortion of the recorded boundary, whereas fast sedimentation processes may in turn require faster scan times. A typical sample channel length is 12 mm, and radial increments of 50 μm are commonly used. Therefore, 240 radial steps are recorded in one scan. Respecting the scan time limit of 60 seconds, allows for 250 ms per step. This sets a basic constraint on the possibilities for accumulating and averaging signals during an acquisition.

Test data were recorded to simulate three types of experiments; slow speed (3000 rpm), mid-speed (30 000 rpm) and high speed (60 000 rpm). The channel timing width, also known as the open-window time, where the sample is available for acquiring signal, is a function of the 2.5° sector geometry of the channel, the radial position in the rotor, the length of the spectrometer entrance slit and the speed of the rotor. The open-window time follows an inverse dependence on the rotor speed. The number of revolutions per step is calculated by dividing the seconds per step by the period of rotation at a given speed. The maximum time available for accumulating signal, or maximum step duration, is then computed from the product of the open-window time and revolutions per step. Appropriate gain settings must be chosen, such that a sufficient pixel response is achieved for a given step duration. Using a 1× PAG setting, 35 000 counts (∼80% pixel saturation) from the pixels at the 530 nm wavelength were selected as a basis of comparison. The three speed simulations and corresponding open-window times, maximum revolutions per step and maximum steps duration are provided in Table 1. The camera trigger signals modulating the gating width of the ICCD were set to reflect the values in Table 1, and the gain subsequently adjusted to provide 35 000 counts.

**Table 1:** Values for optimized iStar ICCD acquisition settings for three rotor speed profiles, and resulting SNR^eff^. Also including comparison values from the Open AUC MWL detector previously reported

Acquisition speed profile				Line – focused illumination						Spot-focused illumination						MWL detector	
Rotor speed RPM	‘Open-window’ μs	Rev per step	Max step duration μs	Gating width μs/acqu.	# Ave.	# Rev int.	Gain (0–4096)	SNR	SNR^eff^	Gating width μs/acqu.	# Ave.	# Rev int.	Gain (0–4096)	SNR	SNR^eff^	# Ave.	SNR^eff^
3000	84	12	1008	168	6	2	2600	59	144.5	168	6	2	1100	153	374.8	5	165.5
30 000	8.5	120	1020	170	6	20	2580	57	139.6	170	6	20	1050	140	342.9	16	296.0
60 000	4.2	250	1050	168	6	40	2580	58	143.5	168	6	40	1100	158	391.0	16	296.0

The simplest illumination architecture is a collimated beam, as described above. However, a collimated beam is shown to provide too weak of a signal to achieve an adequate SNR under all possible acquisition configurations. For example an acquisition set to integrate signal over the maximum step duration of 1000 μs by lowering the gain such that 35 000 counts at 530 nm are recorded, provides a SNR of only 60. Alternatively, increasing the gain such that a shorter integration of signal is collected, and then averaging multiple acquisitions up to the 1000 μs duration limit, provides negligible increase in SNR.

**Fig. 6:**
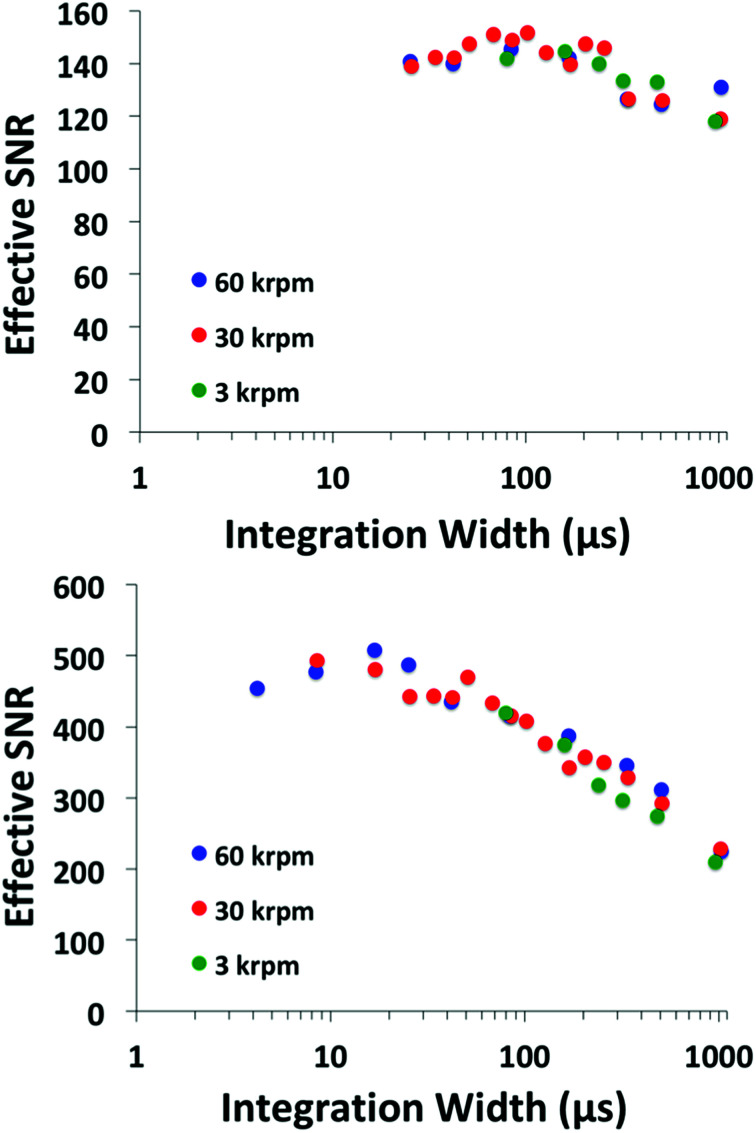
Plots of SNR^eff^*versus* signal integration width, where SNR^eff^ is computed by scaling *N*^1/2^ averages. (top) Line focused illumination, (bottom) spot focused illumination.

The two alternative illumination strategies described above are tested; line-focused and spot-focused illumination. Each illumination design is tested over a series of gain and gating width settings. Higher gain settings require a shorter gating width, and subsequently result in a lower SNR. However, a shorter gating width means that more signals may be averaged within an acquisition. The gating width was adjusted in integer multiples up to the limit of the maximum step duration. At each interval, the intensifier gain was modulated such that 35 000 counts were recorded from the A/D at 530 nm. SNR was then computed as described previously. The SNR values were converted to SNR^eff^ by a *N*^1/2^ consideration of the number of averages possible based on the integer multiples available within the maximum step duration. This allows for a meaningful comparison of the acquisition schemes and optimum settings to be determined. The integration width *versus* SNR^eff^ is illustrated for the line-focused and spot-focused illumination in Fig. 6.

The same trend is observed for all speed profiles. The maximum SNR^eff^ occurs at values of relatively high gain and narrow gating width. However, the conversions to SNR^eff^ by averaging should also take into account the readout time necessary between successive acquisitions, which will be approximately 5 ms for the 2014 × 255 pixel chip. To determine the optimum configuration, another feature of the iStar camera is considered; the so-called Fast Kinetics mode. The Fast Kinetics mode uses the lower un-illuminated portion of the CCD chip as temporary storage, whereby sequential acquisitions are vertically shifted onto the lower CCD array, the entire chip is then read out as a collection of spectra. Because the imaging system only requires 40 pixels to be integrated for the 1 mm field of view, a total of six sets of spectra may be collected on the 255 pixel array in a single acquisition. The vertical shift speed is 6.5 μs, therefore only 260 μs are required to shift each 40 pixel spectrum and may be accomplished within one rotor revolution, even at 60 000 rpm. Therefore the optimum configuration is considered based on six averaged spectra.

The results of the line-focused and spot-focused illumination arrangements are provided in Table 1. It should be noted that the values reported for the Andor spectrometer demonstrate an upper limit of what is possible when used with the X2D2 and tungsten filament illumination system. In practice, additional signal losses from sample cells, and inefficiencies in the imaging optics will further reduce light throughput. Higher power illumination systems should therefore be investigated. The values of SNR^eff^ for the Open AUC MWL detector, as described in Pearson *et al.*, are considered with typical averaging settings and also provided in Table 1.^4^ Note; with the Open AUC MWL detector, the number of possible averages of acquisitions becomes limited by the repetition rate of the xenon flash lamp.

## 4. Conclusions

A new design concept for MWL AUC detector hardware is presented, demonstrating proof of principle test results including spectral sensitivity and SNR analysis. The possibility for two-dimensional imaging is examined, but shown to be unattainable due to the resolution loss when imaging across a grating. Therefore slit-scanning architecture is pursued. The hardware design details and basic imaging performance are outlined in the ESI Fig. 4† and 4, showing how this hardware may be applied in practice. All designs are being published within the framework of the Open AUC initiative, and are freely available from the authors upon request.^11^ The new design offers the possibility for MWL detection down to 190 nm in the UV, much further than previous MWL detectors.^4^ However, broad-spectrum applications require combining acquisitions from multiple gratings. This might enable the future investigation of important deep UV absorbing molecules, such as polysaccharides, without the necessity of labeling. In addition, the spectrometer features easily exchangeable grating options to cover a range of spectral regions with tunable resolution. The SNR performance is probed under various illumination conditions with a deuterium–tungsten light source. Collimated, and line focused illumination are shown to be insufficient for high quality signal acquisitions. Whereas spot focused illumination results in a SNR^eff^ greatly exceeding what is presently possible in MWL AUC detection. These results suggest two possibilities for design strategies going forward. First, a spot focused illumination optical system may be constructed with off-axis parabolic mirrors, and implemented with a scanning assembly, similar to what has been demonstrated previously.^4^ However, this may be difficult to realize within the architecture of the CFA. Second, a more powerful UV/vis light source could be utilized. Laser driven light sources (LDLS) are a promising new possibility in illumination technology offering balanced high brightness UV/vis illumination. At present LDLS systems are prohibitively expensive. We hope the cost of LDLS technology will be reduced in the coming years allowing for integration in this optical system. Implementation of line focused or collimated illumination with a high power lamp is advantageous, as this would negate the requirement of the illumination system scanning together with the detector.

We believe the results presented here demonstrate a promising new direction for MWL AUC absorbance detector technology. Furthermore, we anticipate the findings here to be pertinent for other groups pursuing constant illumination designs in absorbance based AUC detection. In particular, it should be emphasized that shot-noise limited signal quality will also apply to other high sensitivity detector technologies.

## Conflicts of interest

There are no conflicts to declare.

## Supplementary Material

RA-008-C8RA08752K-s001
