# Supplementary material for: ICCD camera technology with constant illumination source and possibilities for application in multiwavelength analytical ultracentrifugation
Source: RSC Adv. 2018 Dec 5;8(71):40655–62. doi: 10.1039/c8ra08752k (PMC9091464; doi:10.1039/c8ra08752k)
Supplement: RA-008-C8RA08752K-s001 [file RA-008-C8RA08752K-s001.pdf]

Electronic Supplementary Material (ESI) for RSC Advances.
This journal is © The Royal Society of Chemistry 2018

ICCD Camera Technology with Constant Illumination Source in Multiwavelength Analytical Ultracentrifugation: Electronic Supplementary Information

Joseph Pearson[a] and Helmut Cölfen [a]

a. University of Konstanz, Physical Chemistry, Department of Chemistry; Universitätsstraße 10, Konstanz 78457 Germany

ESI Table 1; Performance specification comparison of an Ocean Optics USB2000+ Spectrometer and Xenon flash lamp based spectrophotometer system for MWL AUC detection versus an Andor iStar ICCD Shamrock Spectrograph and Deuterium-Tungsten lamp based spectrophotometer system[1-4].

	USB2000 Spectrometer / Xenon Flash Lamp	Andor iStar / Deuterium-Tungsten Lamp
**Significant Advantages**	Small, inexpensive, fits in XL-A vacuum chamber	ICCD allows for gating and signal amplification (both are required for use of a constant illumination source)
Spectral Profile	Large emmission spikes in Vis, limiting dynamic range;	smoother balanced distribution UV through Vis
Spectral range	Dependent on grating, acquistion settings and desired spectral range	UV/Vis, 190 - 780 nm by software selectable grating. Easily exchangable grating turret.
UV	Limited signal at 230 nm, but possible with flash integration. However, fibers are subject to UV transmission loss over time.	A-SR2-GRT-0400-0250 UV grating (190 - 400 nm), 400 l/mm, 250 nm blaze, 1.4 nm resolution
Vis	Excellent visible response up to 650 nm	A-SR2-GRT-0400-0550 Vis grating (400 - 790 nm), 400 l/mm, 550 nm blaze, 1.4 nm resolution
NIR	With Xenon Lamp and XR grating 650 nm, up to 800 nm using high sensitivity mode and neglecting visible signal	Not yet yested with additional NIR gratings. The Gen 2 intensifer is limted to 850 nm
Broad spectra	Not possible to get UV, Vis, and NIR in same acquistion	Dynamically switchable UV/Vis grating options
Vac Issues	spectromter inside; need fibers optical cables	Spectrometer outside vacuum chamber; mirror optics
Repition rate	Limited at high rotor speeds by flash lamp repitition rate	Only dependent on rotor speed
Acquisition width	~2 µs, determined by Xe pulse width	Variable with ns precision; can match channel width of given rotor speed
Lamp power	Very high (illumination optics dependent) over short ~2 µs duration	Much lower than Xe lamp (can compensate by acquistion width adjustment and signal integration)
Detector	Sony ILX511 2048 pixel linear CCD sensor	Andor iStar A-DH320T-25F-04 1024x255 pixel intensified CCD, P46 phosphor & 25 mm 2nd generation photocathode (W)
Pixel Size	14 x 200 µm	26 x 26 µm
Pixel integration	Not possible (can average after in software)	Software selectable, can set to match spectral resolution and image height
Dark noise	(single dark spectrum) 50 counts RMS	Negligible with cooled CCD chip
Overall DNR	Wavelength and sample concentration dependent (limited by Xenon emmission spikes)	*not yet tested
Specified DNR	$2x10^8$(system), 1300:1 (single acquisition)	*not specified by manufacturer
Pixel well depth	*not specified by manufacturer	500,000 e-
Readout rate	2.4 MHz	0.05, 1, 3 or 5 MHz (software selectable)
Read noise	(single dark spectrum) 50 counts RMS	Readout rate dependent; @ 1 MHz; 13 e-
Gain	No gain adjustment option	Software selectable
Sensitivity	*not specified by manufacturer	2 to 10 e-/count provided in documentation
Linearity	0.1 to 1.2 OD	*not yet tested
SNR	250 specified by manufacturer	Dependent on acquisition settings
Illumination	requires Xenon Flash Lamp	Allows for Deuterium-Tungsten Constant Source

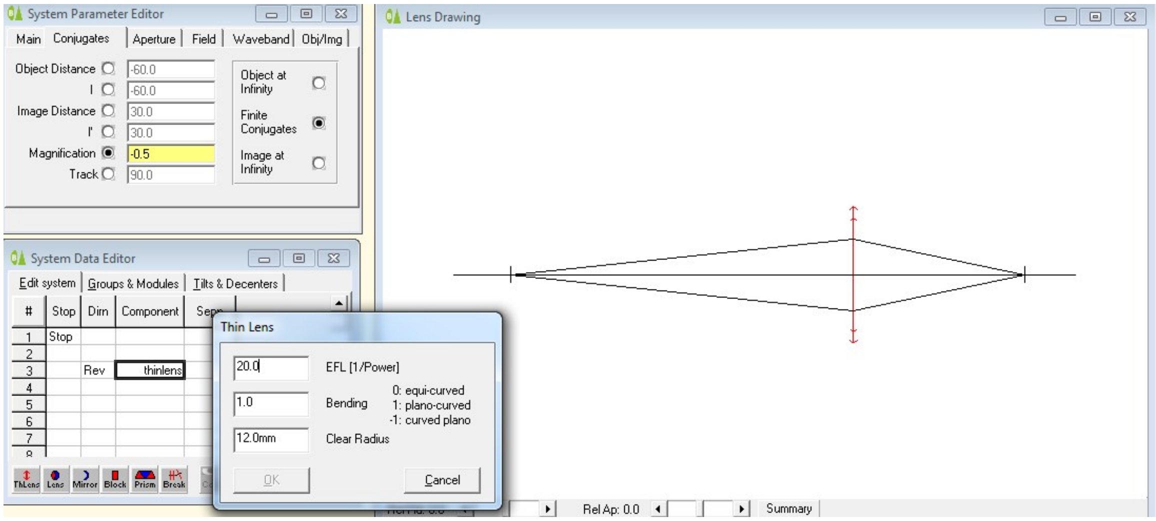

ESI Figure 1; Illumination optics designs, modeled in Winlens 3D Basic from Qioptiq. Screen capture of Winlens 3D model for focusing Tungsten lamp into Hamamatsu X2D2 Deuterium 'see-through' lamp.

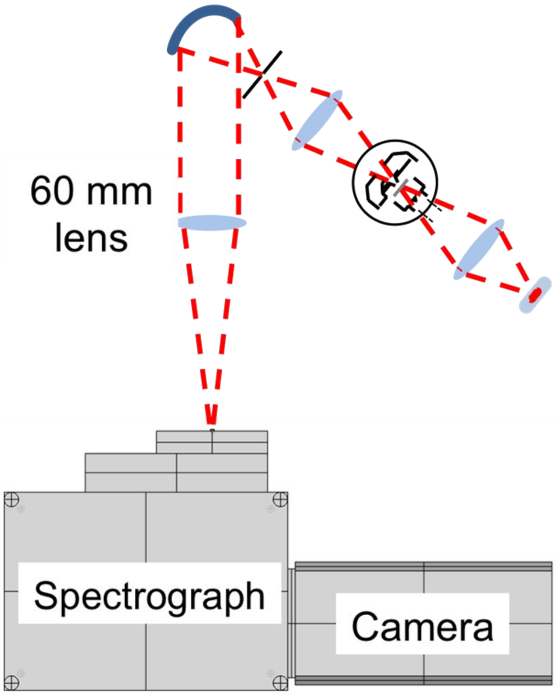

ESI Figure 2; Test setup schematic. The 60 mm focal length lens may be either bi-convex for spot-focused illumination, cylindrical for line-focused illumination, or removed entirely for collimated illumination.

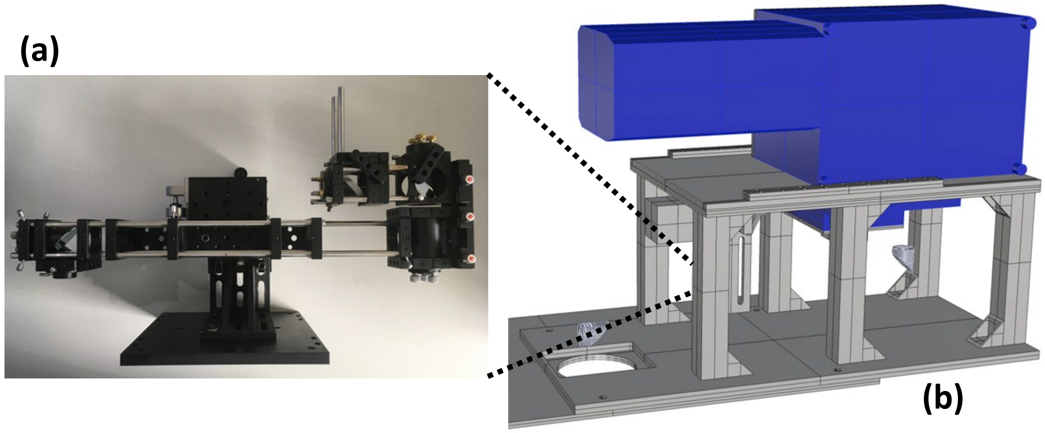

ESI Figure 3; Compact imaging optics are designed based off the concept of the mirror imaging optics published previously, and configured to function within the CFA AUC platform[5, 6]. Off axis parabolic mirrors are selected to accommodate the working distance from the CFA detector platform to the rotor holes. The two identical 152.4 mm focal length off axis mirrors from Thorlabs are arranged with confocal geometry. Additional plane mirrors are included to direct the beam along the necessary trajectory of the spectrometer assembly. A sliding stage was designed to hold the spectrometer system and mount onto the optical table of the CFA. The scanning spectrometer track is designed with high precision Schneeburger (4xRN4-225 Rails and 2xKBN4x31 Roller Cage) linear guides, including hardware to mount a Zaber stepper motor (NM17CD-T4- MC04-HSM8 Motor and X-MCB1-KX13B Controller) for radial positioning. The complete system design is shown in ESI Figure 2, and CAD files are available from the authors upon request. (a) Shows the mirror imaging prototype optics constructed with Linos micro-bench rail system parts, two 152.4 mm off-axis parabolic mirrors, and two plane mirrors from Thorlabs. (b) Shows the design concept for the sliding stage to mount an Andor iStar spectrometer system and mirror imaging optics.

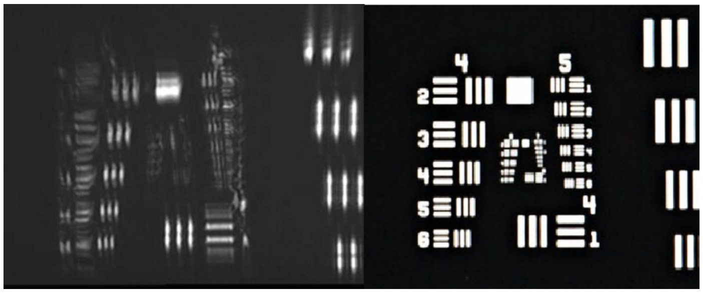

ESI Figure 4; The imaging optics prototype performance is demonstrated by a simple imaging test with a Thorlabs 1951 USAF Resolution Test Target, shown on the left. An ideal Image of 1951 USAF test target from Thorlabs is shown on the right. The image was recorded with a Sony XC-EI30 CCD camera. Determining the resolution from the test target requires identifying the line pairs that may be adequately resolved. In the image, Group 4 - Element 5 is the last resolvable line triplet set. This equates to an optical resolution of 19.7 μm, and fits within the

tolerance of the 25 μm spectrometer slit. It is clear from the image that the resolution is better across the vertical dimension, which corresponds to the dimension parallel with the parent parabola of the mirror, and is the dimension most critical for imaging onto the spectrometer entrance slit.

References

1. Andor, *Andor Technology User's Guide*, 2012.
2. Hamamatsu, *Compact 5 W Xenon Flash Lamp Modules Data Sheet*, 2015.
3. Hamamatsu, *X2D2 High Brightness Deuterium Lamp Data Sheet*, 2018.
4. Ocean-Optics, *USB2000+ Data Sheet*, 2013.
5. J. Pearson, J. Walter, W. Peukert and H. Cölfen, *Anal Chem*, 2017, **90**, 1280-1291.
6. J. Pearson, M. Hofstetter, T. Dekorsy, M. Totzeck and H. Cölfen, *Analyst*, 2018, **143**, 3961–4208.
